# Natural Products from Microalgae with Potential against Alzheimer’s Disease: Sulfolipids Are Potent Glutaminyl Cyclase Inhibitors

**DOI:** 10.3390/md14110203

**Published:** 2016-11-02

**Authors:** Stephanie Hielscher-Michael, Carola Griehl, Mirko Buchholz, Hans-Ulrich Demuth, Norbert Arnold, Ludger A. Wessjohann

**Affiliations:** 1 Group Algae Biotechnology, Department of Applied Biosciences and Process Technology, Anhalt University of Applied Sciences, 06366 Köthen, Germany; stephanie.hielscher-michael@hs-anhalt.de; 2 Department of Bioorganic Chemistry, Leibniz Institute of Plant Biochemistry, 06120 Halle (Saale), Germany; 3 Department of Drug Design and Target Validation, Fraunhofer Institute for Cell Therapy and Immunology IZI, 06120 Halle (Saale), Germany; mirko.buchholz@izi.fraunhofer.de (M.B.); Norbert.Arnold@ipb-halle.de (N.A.)

**Keywords:** glutaminyl cyclase (QC) inhibitor, microalgae, sulfolipids, AD, reverse metabolomics, *Scenedesmus* sp., natural product

## Abstract

In recent years, many new enzymes, like glutaminyl cyclase (QC), could be associated with pathophysiological processes and represent targets for many diseases, so that enzyme-inhibiting properties of natural substances are becoming increasingly important. In different studies, the pathophysiology connection of QC to various diseases including Alzheimer’s disease (AD) was described. Algae are known for the ability to synthesize complex and highly-diverse compounds with specific enzyme inhibition properties. Therefore, we screened different algae species for the presence of QC inhibiting metabolites using a new “Reverse Metabolomics” technique including an Activity-correlation Analysis (AcorA), which is based on the correlation of bioactivities to mass spectral data with the aid of mathematic informatics deconvolution. Thus, three QC inhibiting compounds from microalgae belonging to the family of sulfolipids were identified. The compounds showed a QC inhibition of 81% and 76% at concentrations of 0.25 mg/mL and 0.025 mg/mL, respectively. Thus, for the first time, sulfolipids are identified as QC inhibiting compounds and possess substructures with the required pharmacophore qualities. They represent a new lead structure for QC inhibitors.

## 1. Introduction

Glutaminyl cyclases (QC, EC 2.3.2.5) belong to the class of acyl transferases (EC 2.3.2). These enzymes catalyze the intramolecular cyclization of *N*-terminal l-glutamine residues of peptides and proteins into pyroglutamic acid (5-oxo-prolyl, pGlu*, pE) releasing ammonia, as well as the intramolecular cyclization of *N*-terminal glutamate residues into pyroglutamic acid [1,2,3]. Such a type of post-translational modification stabilizes the peptides and proteins, protects them from proteolytic degradation, and can be important for their biological activity [4,5].

The enzyme QC was first isolated and described by Messer and Ottesen from the latex of the plant *Carica papaya* in 1964 [6]. However, the physiological functions of the plant QC are not completely studied. It was suggested, that this enzyme may plays a role in the plant defense against pathogenic microorganisms [7]. Furthermore, different types of QCs were identified in bacteria, plants and animals [1,2,8,9], as well as in mammalian tissues [10,11,12]. In the latter case, QC is expressed especially in areas of the central nervous system, such as the pituitary, hypothalamus, hippocampus, striatum and exocrine glands like thyroid and thymus [1,2,10]. A number of peptide hormones and chemokines such as Orexin A, gastrin, gonadotropin, TRH, MCP-1 to 4, FPP, fibronectin and neurotensin are substrates of QC.

Although the physiological function of several QC enzymes is still ambiguous, different studies described the pathophysiological connection of human QC to various diseases like arthritis, osteoporosis and Alzheimer’s disease (AD) [13,14]. QC are responsible for the formation of pGlu-modified Aβ peptides in AD, which are more neurotoxic, hydrophobic and resistant to aminopeptidase degradation compared to unmodified Aβ peptides and thus accumulate in AD brains [15,16,17,18,19]. Recent work revealed that the *N*-terminal pGlu-formation is catalyzed by QC not only in vitro but also in vivo [20,21,22,23]. Moreover, QC is detected in neuronal populations with a highly reduced number of synapses and neurons, which are biochemical characteristics of AD. A direct correlation between the overexpression of QC and the vulnerability of neuronal populations could be described [24]. Inhibition of QCs may have therapeutic potential to treat disorders associated with protein aggregation and (neuro) inflammation and thus might be regarded as a therapeutic approach in the treatment of AD [25].

The aim of this study was the identification, characterization and isolation of new QC inhibiting compounds from algae. Preliminary studies showed a positive effect of some algal extracts in QC inhibition. However, all attempts to identify the bioactive principles(s) by the traditional methods, i.e., bio-activity guided isolation or total separation and high-throughput screening of constituents, failed. For the identification of the active constituent in complex extracts this is not uncommon. Therefore, a new technique developed in house (by LAW) termed “reverse metabolomics” was used, in which metabolomics methods are combined with strategies of natural product isolation. The methodology is based on the correlation of chromatographic or spectroscopic profiles of total or partial extracts, especially metabolite profiles, with bioactivity profiles of extracts by chemo-informatic methods (activity correlation analysis, AcorA). This approach enables the direct identification of known and unknown activity-relevant metabolite signals in complex mixtures without their prior separation, and the subsequent directed isolation of the most relevant hit compounds based on this information [26,27].

Therefore, the metabolite profiles obtained by ultra-performance liquid chromatography electrospray ionization mass spectrometry (UPLC-ESI-MS) and direct infusion electrospray ionization Fourier transform ion cyclotron resonance mass spectrometry (FTICR-MS) were correlated with QC inhibiting data.

## 2. Results

The application of the activity correlation analysis (AcorA) for the identification of QC inhibitors from algae necessitates both homologies and variances in the metabolite compositions of the algae extracts, i.e., extracts should be different but also have some common constituents in varied quantities for optimal deconvolution later, referring here specifically to differential mass spectral data and bioactivities among samples. The necessary variations and homologies were achieved by different sources of the biological extracts, using (six) not too distinct but still different, algae species from different classes, families, and genera as well as by harvesting the biomasses at two different growth phases (exponential growth and stationary growth phase). Additional variations were introduced by modifications of the extraction procedure, where the algal biomass of the exponential growth phase (GP) and stationary growth phase (SP) of each species were extracted by two solid-liquid-extraction procedures (Single solvent extraction (s) and multi-step solvent extraction (m)) using n-hexane, methanol and water. A pre-screening of different algae extracts resulting from the solvents n-hexane, methanol and water demonstrates that crude methanolic extracts constitute the most frequent and prominent inhibition properties. Methanol extracts of several algae strains seem to be particularly rich in effective compounds and produce the most promising metabolite profiles [28]. From 24 methanol extracts of the algae *Scenedesmus rubescens*, *Scenedesmus producto-capitatus*, *Scenedesmus accuminatus*, *Scenedesmus pectinatus*, *Tetradesmus wisconsinensis* and *Eustigmatos magnus* from exponential growth phase (GP) and stationary growth phase (SP), 24 chlorophyll-free methanolic solutions were prepared and were selected for correlation analyses at a concentration of 0.2 mg/mL. The results of the QC assay are given in the following Table 1.

A total number of 22 extracts showed QC inhibition in a range of 15% to 72%. The results (Table 1) obtained by the QC-assay were directly correlated with the MS-based metabolite profiles using AcorA [26,27]. The metabolite profiles of the extracts were determined in triplicate by UPLC/ESI-MS and ESI-FTICR-MS both in the positive and negative ion mode. Based on the pre-processed mass spectrometric data and the QC inhibition data, the resulting hit lists from activity correlation analysis were evaluated regarding bioactivity relevant peak clusters (Table 2). Due to the fact that the QC inhibitors were identified by the correlations with the negative ion mode UPLC/ESI-MS and ESI-FTICR-MS data, only these are presented. Comparison of the hit lists from UPLC/(−)ESI-MS and ESI-FTICR-MS, shown in Table 2, after annotation of the MS spectra exhibited a positive correlation of similar activity relevant peak clusters to the bioactivity. The hit list of the UPLC/ESI-MS data in the negative ion mode consisted of 4652 peaks, of which 131 peaks possessed a correlation coefficient >0.6. The hit list of the ESI-FTICR-MS data in the negative ion mode showed only 41 peaks, of which 27 had a correlation coefficient >0.5 and therefore exhibit a positive correlation with the QC inhibition activity. Based on three equal activity relevant peak clusters, compounds **1**–**3** could be identified using AcorA. The first activity relevant compound **1** at *m*/*z* 815.49982 (815.49827) ([M − H]^−^, calcd. *m*/*z* 815.498472 for C_43_H_76_O_12_S) correlates on rank 1 (correlation coefficient 0.75) of the negative ion ESI-FTICR-MS data hit list together with its isotope peaks at *m*/*z* 816.50348 on rank 7 with a correlation coefficient of 0.68. The same compound **1** correlates on rank 4 at *m*/*z* 815.49982 with a correlation coefficient of 0.83 together with its isotope peaks at *m*/*z* 817.6333 on rank 22 with a correlation coefficient of 0.74 in the hit list of the negative ion UPLC/ESI-MS data. A further bioactivity relevant compound **2** at *m*/*z* 817.51617 (817.51596) ([M − H]^−^, calcd. *m*/*z* 817.514122 for C_43_H_78_O_12_S) shows a correlation on rank 13 with a correlation coefficient of 0.63 in the negative ion ESI-FTICR-MS data hit list as well as on rank 8 in the negative UPLC/ESI-MS data hit list with a correlation coefficient of 0.80. In addition, its isotope peaks at *m*/*z* 819.53126 correlate on rank 14 in the negative ion ESI-FTICR-MS hit list and at *m*/*z* 818.5999 with an equal correlation coefficient of 0.80 on rank 9 in the negative ion UPLC/ESI-MS hit list. A compound **3** at *m*/*z* 793.51536 (793.51441) ([M − H]^−^, calcd. *m*/*z* 793.514122 for C_41_H_78_O_12_S) correlates on rank 19 of the negative ion ESI-FTICR-MS data hit list with its isotope peaks at *m*/*z* 794.52123 on rank 9 as well as at *m*/*z* 795.52035 with a correlation coefficient of 0.64 on rank 10.

These identified activity relevant compounds **1**–**3** were further investigated by targeted UPLC/ESI-MS^n^ fragmentation studies. The characteristic fragment ions are listed in Table 3. In their MS^2^ spectra, the fragment ion [b] at *m/z* 537.2 ([M–H–C_16_H_32_O_2_]^−^) could be detected and indicated a loss of a C16:0 fatty acid chains. Furthermore the mass spectral fragmentation of the compounds **1** and **2** shows ions at *m*/*z* 559.2 [a_1_] ([M–H–C_18_H_30_O_2_]^−^) and at *m*/*z* 561.2 [a_2_] ([M–H–C_18_H_32_O_2_]^−^), which indicates the loss of a second fatty acid chain. Moreover, a key ion at *m*/*z* 225 [d] could be detected in the MS^3^ spectra of compounds **1**–**3**. This fragment ion [d] results after loss of the fatty acid chains and a glyceride ester bond and is proposed to be the [M − H]^−^ of C_6_H_9_O_7_S. Furthermore the fragment ions [c] from double loss of C_16/18_ fatty acid moieties at *m*/*z* 283 ([M–H–C_16_H_32_O_2_–C_16_H_32_O_2_]^−^, [M–H–C_16_H_32_O_2_–C_18_H_30_O_2_]^−^, or [M–H–C_16_H_32_O_2_–C_18_H_32_O_2_]^−^) was also present in the MS^3^ spectra of the compounds **1**, **2**, or **3**. Beyond the MS^3^ and MS^4^, fragmentation studies of compounds **1**–**3** represent key ions at *m*/*z* 207 [e] (C_6_H_7_O_6_S^−^), *m*/*z* 165 [f] (C_4_H_5_O_5_S^−^), *m*/*z* 125 [g] (C_6_H_5_O_3_S^−^) and *m*/*z* 81 [h] (HO_3_S^−^).

**Scheme 1:**
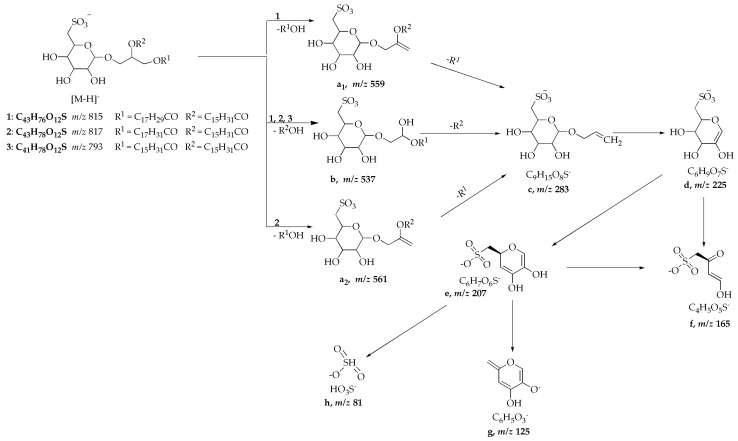
Proposed fragmentation scheme for [M − H]^–^ ions of the compounds **1**–**3** observed in the MS^1^, MS^2^, MS^3^ and MS^4^ experiments in the negative ion mode.

The compounds **1**–**3** show similar fragmentation patterns to the SQDG standard. A common mass spectral fragmentation pattern for the [M − H]^−^ ion of compound **1** (C_43_H_76_O_12_S) at *m*/*z* 815.498472, [M − H]^−^ ion of compound **2** (C_43_H_78_O_12_S) at *m*/*z* 817.51617 and the [M − H]^−^ ion of compound **3** (C_41_H_78_O_12_S) at *m*/*z* 793.51536 is proposed in Scheme 1.

Based on the compared mass spectrometric data (high resolution ESI-FTIC-MS, e.g., comparison of the isotope pattern of the compounds and their theoretically calculated values, postulated fragmentation patterns and database analogy), the compounds **1**–**3** could be identified as 1,2-di-*O*-palmitoyl-3-*O*-(6′-deoxy-6′-sulfo-d-glycopyranosyl)-glycerol (**1**), 1-*O*-palmitoyl-2-*O*-linolenyl-3-*O*-(6′-deoxy-6′-sulfo-d-glucopyranosyl)-glycerol (**2**) and 1-*O*-linolyl-2-*O*-palmitoyl -3-*O*-(6′-deoxy-6′-sulfo-d-glucopyranosyl)-glycerol (**3**), i.e., the compounds are sulfolipids. Targeted UPLC/ESI-MS^n^ fragmentation studies of a SQDG standard (Lipid Products (GB)) shows the molecular ion [M − H]^−^ at *m*/*z* 815 such as compound **1**. In the MS^2^ spectra of the SQDG standard the fragment ion [b] at *m*/*z* 537.2 ([M–H–C_16_H_32_O_2_]^−^) and the fragment ion at *m*/*z* 559.2 [a_1_] ([M–H–C_18_H_30_O_2_]^−^) could be also detected, being caused by the loss of two fatty acid chains. Equally the key ion at *m*/*z* 225 [d] ([M − H]^−^ of C_6_H_9_O_7_S), which represents the sulfoquinovosyl head group—an identification structure element of SQDG—could be detected in the MS^3^ spectra. Sulfoquinovosyldiacylglyceride (1,2-di-*O*-acyl-3-*O*-(6′-deoxy-6′-sulfo-d-glycolpyranosyl)-sn-glycerols) are esterified with two fatty acids. In their mass spectra the neutral losses of the respective fatty acids (palmitic acid, linoleic acid or linolenic acid) with the formation of the characteristic fragment C_6_H_9_O_7_S at *m/z* 225 may be found [29,30,31,32]. The natural SQDGs have an α-anomeric glycoside conformation [33]. An exact assignment of the position of the acyl groups on the glyceride, is not unequivocally possible by mass spectrometric investigations. Taking this into account [34], possibly the higher peak intensity of fragment ion [b] at *m/z* 537.2 in comparison to fragment ion [a] suggests the position of the palmitic acid to be at the sn-2-postion. Based on this and other references the positions postulated in the literature are assumed also here [29,35,36,37]. Thus for compound **1** at sn-1-position linolenic acid (C18:3) and at sn-2-position palmitic acid (C16:0), for compound **2** at sn-1-position linoleic acid (C18:2) and at sn-2-position palmitic acid (C16:0) and for compound **3** at sn-1-position and sn-2-position palmitic acid (C16:0) are proposed.

Because reverse metabolomics only gives the most likely correlation, a causal relationship of compound and effect has to be proven. To confirm an inhibitory effect against the QC, of the sulfolipids **1**–**3,** it was necessary to isolate them and to examine them in detail. For this purpose, the methanolic extract of *Sc. accuminatus* eSP was selected and the sulfolipids were isolated using NH_2_ cartridges (SPE) [36]. 25 mg of methanolic extract of *Sc. accuminatus* eSP was added to NH_2_-conditioned cartridges and a two-step elution was conducted. Subsequently, a Folch wash was carried out to eliminate the salts, like ammonium acetate, and to transfer the sulfolipids into the organic methanol/dichloromethane phase. The sulfolipids thus obtained (6.5 mg) were investigated by UPLC-MS. For reference, a SQDG standard (Lipid Products) was used. The mass spectrometric analysis of the sulfolipids by UPLC-MS in the negative ion mode showed a characteristic base peak at RT of 9.9 min in the chromatogram. The MS^1^ confirmed molecular ions [M − H]^−^ at *m*/*z* 794 and 815 is congruent with sulfolipids **3** and **1**. These and the SQDG standard were tested at two concentrations in the QC assay. Both, isolated sulfolipids and the SQDG standard, were able to inhibit QC at concentrations of 0.25 mg/mL and 0.025 mg/mL with 81%/76% and 77%/76%, respectively. The results of the QC assay confirmed that these sulfolipids act as QC inhibitors and thus have potential as lead compounds against diseases associated with QC, namely Alzheimer’s disease.

## 3. Discussion

The reverse metabolomics approach with AcorA was applied to identify QC inhibiting metabolites produced by different algae strains. The applied method allowed the identification and characterization directly from complex crude extracts without further purification steps. It is strongly suggested, that the identified QC inhibitors are sulfolipids, which are already identified from various microalgae (mainly Rhodophyta) like *Porphyridium purpureum* [35], *Heterosigma carterae*, *Phaeodactylum tricorntum* [37], *Heterosigma caterae* [38], and *Pavlova lutheri* [39], as well as from the macroalgae *Gigartina tenella* [40] and *Caulerpa racemosa* [41]. Furthermore, the occurrence of sulfolipids is known from cyanobacteria e.g., *Lyngbya lagerheimii* [42], *Oscillatoria raoi* [43], *Arthrospira platensis*, *Nostoc punctiforme*, *Scytonema hofmanni* [36], and from spinach (*Spinacia oleeracea)* [44]. In photosynthetic organisms, SQDGs are important compounds of the thylakoid membrane and they are necessary for the unrestricted function of the photosystem II [45,46,47,48,49,50,51,52]. Sulfolipids demonstrate a broad activity spectrum like immunosuppressive [43] and antiviral effects (e.g., against HIV) [36,42,53]. Furthermore, antineoplastic effects and inhibitory activity of the enzymatic activities of DNA polymerases pol α and pol β as well as the α-glucosidase and the caspase are known [40,54,55,56,57]. Brahmi et al. detected a telomerase inhibitory activity (anticarcinogenic) of SQDGs isolated from the cyanobacterium *Microcystis aeruguinosa* [58]. In addition, anti-inflammatory activity and anti-proliferative effects on various human (cancer) cell lines are described [59,60,61,62,63,64] as well as a prophylactic effect against *Mycobacterium tuberculosum* infection is known [44]. As part of a patent, the effect of sulfolipids in inflammatory skin diseases, especially psoriasis is described [65]. In the present study, we could demonstrate for the first time that SQDGs exhibit QC inhibitory effects. The new activity for this compound class could be elucidated by application of AcorA, a new chemoinformatic method which allows the direct identification of known and unknown activity-relevant metabolites and their spectroscopic clusters in complex mixtures like algal crude extracts. The evidence for an actual QC inhibition of sulfolipids (**1**–**3**) could be provided through a successful isolation with subsequent testing of QC inhibition.

Previously known QC inhibitors are primarily described in patents of the company Probiodrug AG. The application of QC inhibitors for the treatment of Alzheimer’s disease has proven to be successful in different transgenic animal models [20,66,67]. One competitive QC inhibitor PQ912 for the treatment of Alzheimer’s disease successfully completed clinical trial phase 1 [68] and currently is tested in clinical trial phase 2 [23]. All known QC inhibitors were synthesized or achieved by structure based design and bioisosteric replacement as well as homology modeling. In publications by Buchholz et al., 2006, 2009 [69,70] studies of substructures of QC inhibitors were performed. From this it can be deduced that for QC inhibitors following substructures with pharmacophore characteristics are necessary:

- Metal binding group (MBG)
- Flexible linker with minimum length (propyl-linker)
- Core structure (scaffold) decorated with functional groups at certain positions e.g., 3,4-dimethoxyphenylthiourea, which additionally can form hydrogen bonds and lipophilic interactions within the enzyme pocket.

**Figure 1:**
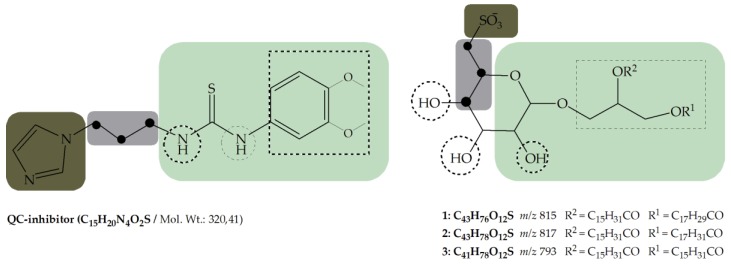
Comparison of the structural characteristics of the QC inhibitor 1-(3-(1H-imidazol-1-yl)propyl)-3-(3,4-dimethoxyphenyl)thio-urea with the QC inhibiting sulfolipids (**1**–**3**). [brown box: metal binding group; grey box: flexible linker; green box: scaffold incl. decoration].

The QC inhibiting sulfolipids (**1**–**3**) have suitable structural characteristics for a QC inhibitor. In Figure 1, the structures of a known QC inhibitor (**4**) and of the sulfolipids are compared.

As shown in Figure 1, the identified sulfolipids in principal provide structural characterizations (substructures) suitable for known QC inhibitors. The negatively charged sulfonate group at the 6-hydroxyl position of the glucose probably acts as a metal binding group. Of course, the polyhydroxy elements may also bound to the metal ion; the ether portion acts as a linker in this very preliminary comparison. However, the assumption depicted in Figure 1 could be enforced by docking studies (not shown). Thus the sulfonate group suggests itself as a metal binding group, not known before for QC inhibitors. The glucose probably acts as core structure (scaffold). The hydroxyl groups may acting as hydrogen acceptors and donors and allow directed interactions in the active site of the enzyme. Starting from the metal binding group it links the scaffold and has an optimal length of 3 methylene units to the 5-hydroxy group of glucose. The necessary flexible linker between the metal binding group and the core structure is also present in the sulfolipid structure. The QC inhibitor **4** has an even more flexible linker with a length of three methylene units to the NH group of the core structure (N1 of the thiourea moiety). The 5-hydroxy group, as well as the NH group, according to the models, will probably act as hydrogen bond donors. By reason of the experimental proof, supported by substructures with somewhat similar pharmacophore qualities, they present a new lead structure for QC inhibitors.

In summary sulfolipids share common, necessary substructures with pharmacophore characteristics of known QC inhibitors and they may be an initial point in developing new QC inhibitors.

## 4. Materials and Methods

### 4.1. Cultivation of the Microalgae

The microalgae strains *Scenedesmus rubescens* (SAG 5.95), *Scenedesmus producto-capitatus* (SAG 21.81), *Scenedesmus accuminatus* (SAG 38.81), *Scenedesmus pectinatus* (SAG 2003), *Tetradesmus wisconsinensis* (SAG 3.99), and *Eustigmatos magnus* (SAG 36.89) were originally purchased from the culture collection of Göttingen University Germany (SAG). The algae were cultivated by using Setlik medium (2.02 g/L KNO_3_, 0.34 g/L KH_2_PO_4_, 0.99 g/L MgSO_4_·7H_2_O, 0.0185 g/L Fe-EDTA, 0.01 g/L Ca(NO_3_)_2_∙4H_2_O, 0.00309 g/L H_3_BO_3_, 0.0012 g/L MnSO_4_∙4H_2_O, 0.0014 g/L CoSO_4_, 0.00124 g/L CuSO_4_·5H_2_O, 0.00143 g/L ZnSO_4_, and 0.00184 g/L (NH_4_)_6_Mo_7_O_24_∙4H_2_O) in a 100 L tubular photobioreactor (IGV GmbH, Nuthetal, Germany) 100 GS/PL with constant illumination of a photon flux density of 90 μmol/m^2^·s (<OD 20) respectively 150 μmol/m^2^·s (>OD 20) at pH 7. The pH value was regulated by the addition of CO_2_.

The harvests of biomass of the growth phase were carried out between day 7–10 by centrifugation. For obtaining the biomass of the stationary growth phase new Setlik medium was applied to the rest of the algae culture after harvesting and the culturing continued (fed-batch-method). After reaching the stationary phase (SP), which was achieved between 9 and 14 days depending from the alga, the biomass was harvested by centrifugation.

### 4.2. Preparation of Crude Extracts

The freeze-dried algal biomasses of the exponential growth phase (GP) and stationary growth phase (SP) of each algae species were extracted by two solid-liquid-extraction procedures (single solvent extraction (s) and multi-step solvent extraction (m)).

In the first extraction procedure (s) each biomass (10 g) was ground with mortar and pestle using sea sand (biomass:sea sand = 1:2) and extracted triply with 600 mL each of methanol. In the second extraction procedure (m), each ground and with n-hexane pre-extracted biomass (10 g), was extracted triply with each 600 mL of methanol. The methanol extracts of both procedures were concentrated to dryness in a rotary evaporator under reduced pressure and re-dissolved with methanol to a defined concentration [28].

### 4.3. Sample Preparation

Chromabond SA-cartridges (Macherey & Nagel, Düren, Germany) were used to remove chlorophyll from the crude extracts (Macherey & Nagel Application-No.: 300010). The resulting pre-cleaned extracts were evaporated to dryness and redissolved in methanol followed by a solid phase extraction on Chromabond C18ec-cartridge (Macherey & Nagel) using methanol as a solvent. The obtained processed extracts were evaporated to dryness and dissolved in methanol for assaying and mass spectrometry measurements.

SQDG standard were purchased from Lipid Products (Nutfield, Redhill, UK).

### 4.4. Glutaminyl Cyclase (QC) Inhibition Assay

The determination of the catalytic QC-enzyme activity was accomplished by utilization of the fluorogenic substrate Gln-AMC (*N*-glutaminyl-7-amino-4-methyl-coumarin) and the supporting enzyme pyroglutaminyl aminopeptidase (pGAP), as well as purified human QC.

QC cyclizes Gln-AMC to pGlu-AMC which serves in a second step as substrate for pGAP. The removal of AMC from pGlu-AMC is than detected at λ_ex_ = 380 nm; λ_em_ = 460 nm [71].

In this coupled optical assay the increase of the free AMC was continuously analyzed for more than 12 min at 30 °C. The assay was conducted in microtiter plates by adding 100 μL 0.25 mM substrate, 50 μL 0.2 mg·mL^−1^ extract, 25 μL supporting enzyme pGAP and 25 μL QC with a final volume of 250 μL per well. Because of the QC pH value dependency, 50 μL Tris buffer (0.1 M; pH 8) was added. The exact procedure was published by Schilling et al. [72,73].

All assay investigations were done in triplicate and if inhibition activities were more than 20% vs. control, it was classified as QC-active.

An influence of an inhibition of the supporting enzyme pyroglutaminyl aminopeptidase (pGAP) could be excluded by investigations.

### 4.5. Mass Spectrometry

#### 4.5.1. UPLC/ESI-MS

The mass spectrometry analysis of the methanol crude extracts were obtained by an Acquity UPLC-system (Waters GmbH, Eschborn, Germany) equipped with an ion trap (LCQ Deca XP MAX, Thermo Finnigan, Thermo Fisher Scientic Inc., San Jose, CA, USA). The ionization was effected by electro spray ionization (4 kV, 275 °C, 27 V, nitrogen flow rate 35–40 arb. units). The separation on a RP-18 HSS T3 1.8 μm, 1.0 × 100 mm (PartNo: 186003536, Serial No.: 01183023815308; Waters) column were accomplished by using a gradient system starting from water/acetonitrile 95:5 (each of them containing 0.2% CH_3_COOH) to 100% acetonitrile within 7.5 min. After continuous flow of 100% acetonitrile to 8.2 min the solvent gradient system of water/acetonitrile 95:5 was reached at 12 min. The measurements were done at a flow rate of 0.150 mL/min and a column temperature of 40 °C. The sample was introduced with partial injection in needle overfill modus and with an injection volume of 1 μL. ESI-MS spectra were acquired in negative and positive ion electrospray ionization mode by scanning over the *m*/*z* range 100–1000 Da in triplicate. Data dependent MS^2^ and MS^3^ experiments were performed by selection of the ions of the highest intensity, whereas the intensity had to be larger than 105 a.i., and using a normalized collision energy of 35% or 45% in negative ion mode and 35% in the positive ion mode (activation Q: 0.250; activation time: 30.0 msec). Isolation width was set at ±2 Da. The data were evaluated by the software Xcalibur 2.0 and 2.0.7 (Thermo Scientific, Waltham, MA, USA).

#### 4.5.2. ESI-FTICR MS

The high resolution ESI mass spectra of the extracts were obtained from a Bruker Apex III Fourier transform ion cyclotron resonance (FTICR) mass spectrometer (Bruker Daltonics, Billerica, MA, USA) equipped with an Infinity™ cell, a 7.0 Tesla superconducting magnet (Bruker, Karlsruhe, Germany), an RF-only hexapole ion guide and an external APOLLO electrospray ion source (Agilent, off axis spray, voltages: endplate, 3.700 V; capillary, −4.200 V; capillary exit, 100 V; skimmer 1, 15.0 V; skimmer 2, 10.0 V). Nitrogen was used as drying gas at 150 °C. The sample solutions were introduced continuously via a syringe pump with a flow rate of 120 μL·h^−1^. All data were acquired with 512 k data points and zero filled to 2048 k by averaging 32 scans. The XMASS Software (Bruker, Version 6.1.2) was used for evaluating the data.

### 4.6. MS Data Processing and Activity Correlation Analysis (AcorA)

The ESI-FTICR MS files were converted to CDF format (Thermo) whereas the UPLC-MS files converted to mzXML format using ProteoWizard. The software-package XCMS was implemented in R (version 2.10.0, https://www.r-project.org/). Data were processed using XCMS with the following parameters for peakpicking and alignment: method = “MSV”, SNR.method = “data.mean”, winSize.noise = 500, peakThr = 80,000, snthresh = 3, amp.Th = 0.005, scales = c (1, seq(7,9,3)); minsamp = 2, mzppm = 7 for FT-ICR-MS and method = “centWave”, mzdiff = 0.3, peakwidth = c (5, 12), snthr = snthr, verbose.columns = F, prefilter = c (2, 500); minsamp = 2, bw = 5, mzwid = 1 for UPLC-MS. The data output effected as aligned data matrix, which used for correlation of the bioactivity data and peak intensities using Spearman rang correlation coefficient (α = 5%). The results were viewed as tables in Excel (Microsoft) and plots in PDF (Adobe).

### 4.7. Isolation of Sulfolipids

Sulfolipids were purified with aminopropyl modified silica gel cartridges (NH_2_-cartridges; Macherey & Nagel, Düren, Germany). These were pre-conditioned in several steps with 2 mL methanol, 2 mL water, 4 mL 0.1 M hydrochloric acid (incubation for 1 h), 2 mL water, 2 mL methanol, 2 mL dichloromethane/isopropanol/methanol (15:30:50; *v*/*v*/*v*). Samples have been dissolved in dichloromethane/methanol (1:1; *v*/*v*) at a concentration of 20 mg to be put on the cartridge. Subsequently, a two-step elution was accomplished. In the first step unloaded compounds were eluted with 9 ml dichloromethane/isopropanol/methanol 15:30:50 (*v*/*v*/*v*) and discarded. The elution of sulfolipids was realized in a second step with 5 mL dichloromethane/acetonitrile/isopropanol/methanol/0.1 M aqueous NH_4_OAc (10:10:10:50:15; *v*/*v*/*v*/*v*/*v*) [36].

The obtained sulfolipids were evaporated to dryness and subsequently desalted. Therefore dichloromethane/methanol/0.1 M NaOAc-Puffer pH = 4.0 (8:4:3, *v*/*v*/*v*) was added and vigorously shaken. After overnight incubation at 4 °C, a two-phase system was formed, where the sulfolipids accumulate in the lower lipophilic phase. This lower sulfolipid phase was removed with a Pasteur pipette, transferred to a round bottom flask, and concentrated to dryness using a vacuum rotary evaporator.

## 5. Conclusions

Algae extracts of different species were investigated regarding their QC inhibiting activity. Since traditional methods were unsuccessful to identify the active principle, a new approach called Reverse Metabolomics with Activity-correlation Analysis (AcorA) was carried out, as this method does allow the identification of active principles in complex mixtures, and even additive or synergistic relationships can be discovered. Thereby a direct correlation of the mass spectrometry metabolite profiles (fingerprints) of the UPLC/ESI-MS or ESI-FTICR-MS data with the QC inhibition value of each extract was applied. The correlation analysis of the UPLC/ESI-MS and ESI-FTICR-MS data showed similar metabolite peak clusters for three significantly correlated compounds in the negative ion mode. By additional targeted mass spectrometric fragmentation analyses, the correlated compounds could be structurally characterized directly from the complex extracts without purification. Based on these results, the QC inhibiting compounds were specifically isolated and, by comparison with a reference compound, verified as sulfolipids, specifically suggested to be 1,2-di-*O*-palmitoyl-3-*O*-(6′-deoxy-6′-sulfo-d-glycopyranosyl)-glycerol (**1**), 1-*O*-palmitoyl-2-*O*-linolenyl-3-*O*-(6′-deoxy-6′-sulfo-d-glucopyranosyl)-glycerol (**2**), and 1-*O*-linolyl-2-*O*-palmitoyl-3-*O*-(6′-deoxy-6′-sulfo-d-glucopyranosyl)-glycerol (**3**). The evidence for an actual QC inhibition of sulfolipids (**1**–**3**) could be provided through their successful isolation with subsequent testing of QC inhibition. Thus, for the first time QC inhibitors from algae were identified, characterized and isolated. Likewise, for the first time the inhibitory effect of sulfolipids from algae against QC was demonstrated. Until now, no natural product with QC inhibitor activity is known, thus this is the first description of a natural QC inhibitor, present in algae species.

## Figures and Tables

**Table 1:** QC inhibition activities [%] of the chlorophyll-free methanol extracts of 6 different algae species harvested at two growth phases (exponential growth phase (GP) and stationary growth phase (SP)) by two extraction procedures (s = single solvent extraction, and m = multi-step solvent extraction).

Algae Extract	Inhibition of QC Enzyme Activity [%] * c_extract_ = 0.2 mg/mL	Algae Extract	Inhibition of QC Enzyme Activity[%] * c_extract_ = 0.2 mg/mL
*Sc. producto-capitatus* sGP	59	*Sc. pectinatus* mGP	32
*Sc. producto-capitatus* mGP	24	*Sc. pectinatu*s sGP	43
*Sc. producto-capitatus* sSP	15	*Sc. pectinatu*s mSP	21
*Sc. producto-capitatus* mSP	35	*Sc. pectinatus* sSP	63
*Sc. rubescens* sGP	65	*Tetradesmus wiscon.* mGP	39
*Sc. rubescens* mGP	23	*Tetradesmus wiscon.* sGP	72
*Sc. rubescens* sSP	56	*Tetradesmus wiscon*. mSP	19
*Sc. rubescens* mSP	22	*Tetradesmus wiscon.* sSP	16
*Sc. accuminatus* sGP	44	*Eustigmatos magnus* mSP	0
*Sc. accuminatus* mGP	26	*Eustigmatos magnus* sSP	0
*Sc. accuminatus* sSP	57	*Eustigmatos magnus* mGP	56
*Sc. accuminatus* mSP	22	*Eustigmatos magnus* sGP	61

* Inhibition of QC enzyme activity = QC activity without inhibitor/extract − residual QC activity after measurement; (QC enzyme activity [%] − residual activity [%]).

**Table 2:** Hit list of the top ranked (depending on Spearman’s rank correlation coefficient *r*_s_) activity relevant peak clusters based on UPLC/ESI-MS data and ESI-FTICR-MS data both in the negative ion mode correlated with the QC inhibition activity.

UPLC/ESI-MS					FTICR-ESI-MS						
Rank	*r*_s_	[M − H]^−^ *(m/z)*	RT min	Possible Compound	Rank	*r*_s_	[M − H]^−^ *(m/z)*	Possible Compound	Elemental Composition	Calcd. [M − H]^−^ (*m/z*)	DBE
					1.	0.75	815.49982 815.49827 *	1	C_43_H_76_O_12_S	815.498472 *	5.0
4.	0.83	815.7333	7.22	1							
					7.	0.68	816.50348	Isotope p. *m/z* 815.50			
8.	0.80	817.6666	7.70	2							
9.	0.80	818.5999	7.70	Isotope p. *m/z* 817.67	9.	0.64	794.52123	Isotope p. *m/z* 793.52			
					10.	0.64	795.52035	Isotope p. *m/z* 793.52			
					13.	0.63	817.51617 817.51596 *	2	C_43_H_78_O_12_S	817.514122*	4.0
					14.	0.63	819.53126	Isotope p. *m/z* 817.52			
15.	0.75	794.7333	8.86	Isotope p. *m/z* 793.73							
					19.	0.56	793.51536 793.51441 *	3	C_41_H_78_O_12_S	793.514122*	2.0
22.	0.74	817.6333	7.22	Isotope p. *m/z* 815.73							

* The elemental formulas were calculated based on the elemental composition of high-resolution FT-ICR-MS data out of *m*/*z* by repeated direct measurement of the extracts.

**Table 3:** Negative ion UPLC/ESI-MS^n^ data of compounds **1**–**3** and a SQDG standard (Lipid Products (GB)).

Compound	[M − H]^−^ (*m/z*)	Scan Mode [*m/z*]	*m/z* Relative Intensity (%) [Fragment Ion Schema 1]
**1**	815	MS^2^ [815] MS^3^ [815 → 537]	537 ([b], 100), 559 ([a_1_], 38) 225 ([d], 100), 207 ([e], 7), 165 ([f], 12), 283 ([c], 26)
**2**	817	MS^2^ [817] MS^3^ [817 → 537]	537 ([b]), 100), 561 ([a_2_], 32) 225 ([d]), 100), 165 ([f], 30), 207 ([e], 9), 283 ([c], 3)
**3**	793	MS^2^ [793] MS^3^ [793 → 537] MS^4^ [793 → 537 → 225]	537 ([b], 100), 225 ([d], 20) 283 ([c], 42), 207 ([e], 53), 225 ([d], 100) 207 ([e], 52), 165 ([f], 60), 125 ([g], 100), 81 ([h], 27)
SQDG standard	815	MS^2^ [815] cMS^3^ [815 → 537] MS^3^ [815 → 559]	537 ([b], 100), 559 ([a_1_], 90) 225 ([d], 100), 165 ([f], 58), 207, ([e],30) 225 ([d], 100), 283 ([c], 38)

## Abbreviations

The following abbreviations are used in this manuscript:

AcorA	Activity-correlation Analysis
AChE	acetylcholine esterase
APP	amyloid precursor protein
AD	Alzheimer’s disease
BACE-1	Beta-secretase 1; beta-site amyloid precursor protein cleaving enzyme 1
calcd.	calculated
CH_3_COOH	acetic acid
ESI-FTICR MS	electrospray ionization Fourier transform ion cyclotron mass spectrometry
Gln-AMC	*N*-glutaminyl-7-amino-4-methyl-coumarin
GP	exponential growth phase
Isotope p.	isotope peak
MBG	metal binding group
NaOAc	sodium acetat
NH_4_OAc	ammonium acetate
pGAP	pyroglutaminyl aminopeptidase
pGlu	pyroglutamyl
r_s_	Spearman’s rank correlation coefficient
RT	retention time
SP	stationary growth phase
QC	glutaminyl cyclase
UPLC-MS	Ultra performance liquid chromatography mass spectrometry
